# Alagille syndrome due to a novel *JAG1* mutation in a Chinese pediatric patient: A case report

**DOI:** 10.1097/MD.0000000000044908

**Published:** 2025-10-17

**Authors:** Zongdan Zhang, Xuezu Zhang, Chuangfeng He

**Affiliations:** a Department of Disease Prevention and Control, Baoshan People’s Hospital, Yunnan, China; b Department of Pediatrics, Lincang People’s Hospital, Yunnan, China; c Department of Pediatric Internal Medicine, Chongqing YouYouBaoBei Women’s and Children’s Hospital, Children’s Hospital Medical Consortium Affiliated to Chongqing Medical University, Chongqing, China.

**Keywords:** a novel mutation, Alagille syndrome, chronic cholestasis, *JAG1* gene, pulmonary artery stenosis, whole-exome sequencing

## Abstract

**Rationale:**

Alagille syndrome (ALGS) is a multisystem autosomal dominant disease with variable expressivity and phenotypic penetrance, characterized by abnormalities in liver, heart, skeleton, eyes, kidneys, face, and vasculature. Mutations in *JAG1* are the major genetic cause of ALGS. Here, we illustrated the clinical and genetic characteristics of a pediatric ALGS case caused by a novel *JAG1* mutation, aiming to strengthen clinicians’ awareness and understanding of ALGS at clinical and molecular levels.

**Patient concerns:**

A 5-year-old Chinese female presented with suspected ALGS clinical phenotypes including chronic cholestasis, liver dysfunction, and pulmonary artery stenosis.

**Diagnoses:**

A novel heterozygous missense mutation (c.3112C > G, p.L1038V) in the exon 25 of the *JAG1* gene was identified in this patient via whole-exome sequencing. Nucleotide sequence conservation analysis showed that the novel *JAG1* c.3112C > G mutation was located in evolutionarily conserved region, suggesting its potential importance to the function of JAGGED1 protein encoded by *JAG1*. Subsequently, pathogenicity of this novel mutation was further confirmed by flow cytometry which demonstrated a defective expression of wild-type JAGGED1 protein. Combining the clinical features with genetic findings, this patient was eventually diagnosed as ALGS.

**Interventions:**

Patient received supportive treatments, including blood transfusion, vitamin K1 supplementation, and the application of liver-protection drugs.

**Outcomes:**

This patient developed liver failure during hospitalization. Regrettably, the guardians gave up further therapies (blood purification therapy and liver transplantation) for the patient and asked to be discharged. Soon after, the patient died at home due to deterioration of her condition.

**Lessons:**

In the present study, a novel pathogenic *JAG1* mutation was identified using whole-exome sequencing in a Chinese patient with ALGS phenotype, expanding the mutational spectrum of *JAG1* gene.

## 1. Introduction

Alagille syndrome (ALGS; OMIM#118450) is a rare autosomal dominant multisystem disease with an estimated incidence of 1 per 30,000 live births and is characterized by various degrees of abnormalities in liver, heart, skeleton, eyes, kidneys, face, and vasculature.^[1–3]^ The first description of ALGS was reported by Daniel Alagille et al in 1962 and the initial diagnostic criteria of ALGS were also defined by him in 1987 as follows: the presence of intrahepatic bile duct paucity and at least 3 out of the 5 major clinical characteristics including chronic cholestasis, characteristic facial features, structural cardiac disease, skeletal abnormalities and ocular defects.^[4,5]^ However, due to the lack of molecular genetic techniques in the past, the accurate diagnosis of ALGS was difficult. Before the era of molecular diagnosis, the incidence of ALGS was misestimated as 1 per 70,000 live births by relying solely on classic clinical features and this figure was significantly lower than the true incidence mentioned above (1 per 30,000 live births).^[6]^

**Figure 1.**
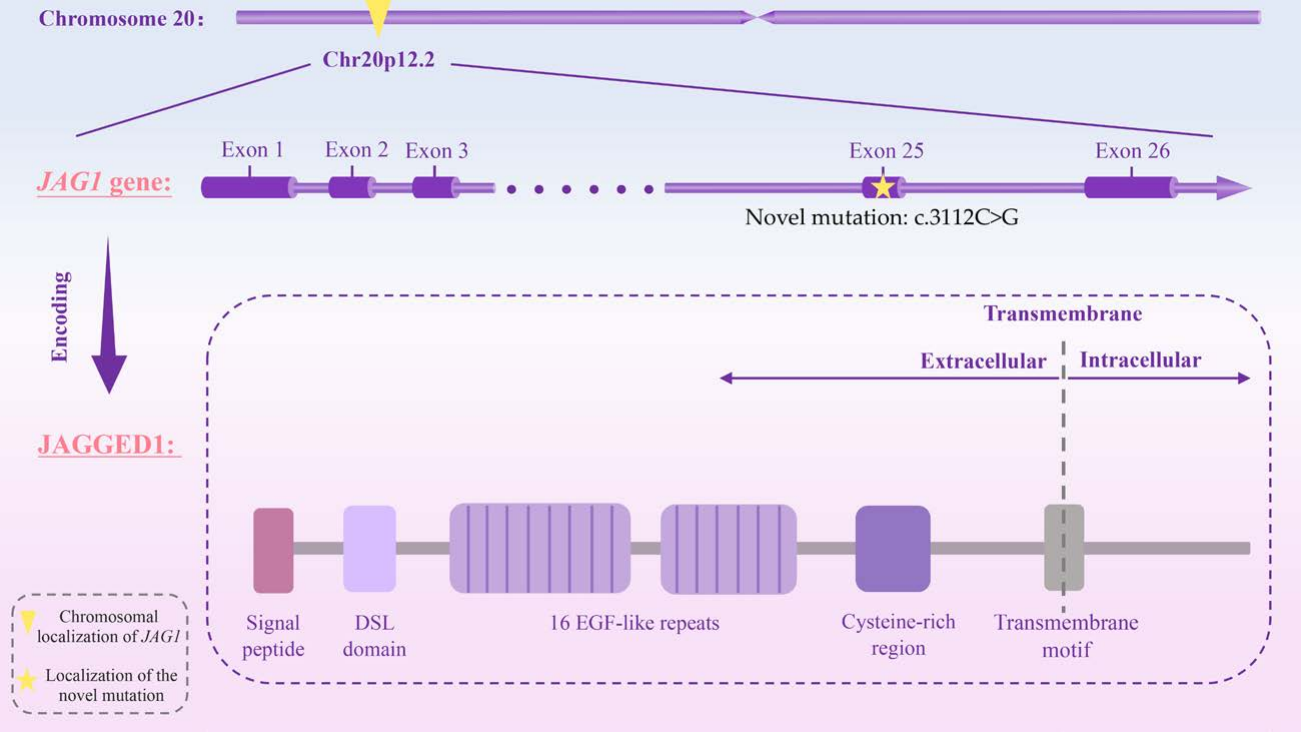
The localization of *JAG1* gene and the structure of its encoded protein JAGGED1. DSL = delta-serate-lag2, EGF = epidermal growth factor.

Encouragingly, after the first report in 1986 identified a deletion in chromosome 20p12 in a child with the classical ALGS phenotype, mutations in the *JAG1* gene and the *NOTCH2* gene were subsequently linked to the molecular pathogenesis of ALGS in 1997 and 2006, respectively.^[1,7]^ Among those mutations found in the reported cases of ALGS, mutations in the *JAG1* gene, a 3657-nt long gene located on chromosome 20p12.2 and comprised of 26 exons (Fig. 1), were by far the most common causes, accounting for over 90% of all ALGS.^[8]^ The JAGGED1 protein encoded by the *JAG1* gene is the cell surface ligand of the Notch signaling pathway, which binds and interacts with Notch transmembrane receptors, introducing γ-secretase-dependent cleavage of the Notch receptor, the release of the Notch intracellular domain, and the translocation of the Notch intracellular domain into the nucleus, ultimately driving the expression of downstream target genes. The receptor–ligand pairs generated by JAGGED1 and Notch receptors are crucial parts of the evolutionarily conserved Notch signaling pathway, which plays a significant role in regulating cell fate during the development of multiple types of cells.^[9,10]^ JAGGED1^[10]^ consists of a small intracellular domain, a transmembrane domain, and a large extracellular domain, while reported *JAG1* mutations are all found in the extracellular domain, which is the portion of JAGGED1 that directly binds to Notch receptors and contains multiple functional motifs crucial for the interaction, including an N terminal signal peptide, a highly conserved delta-serate-lag2 domain, 16 epidermal growth factor-like repeats, and a cysteine-rich region (Fig. 1). Therefore, mutations occurring in *JAG1* likely alter the initiation of normal signal transduction downstream of Notch, inducing abnormal proliferation and differentiation of cells, which may eventually lead to ALGS-related clinical presentations. Although the above findings in genetics substantially advanced our understanding of the molecular basis of ALGS and facilitated the precise diagnosis of this disease, approximately 2% to 4% of clinically diagnosed ALGS cases still do not have an identified mutation.^[11]^ Furthermore, establishing an exact relationship between genotype and phenotype remains a challenge due to the high complexity and heterogeneity of AGLS.

In this report, we elucidated the molecular and phenotypic features of an ALGS patient with a novel heterozygous missense *JAG1* mutation not yet described in the available literature or public genetic databases. This work was approved by the Ethics Committee of Baoshan People’s Hospital (approval number: 2024-05-129-K01) and written informed consent was obtained from the guardian of the proband.

## 2. Case report

**Figure 2.**
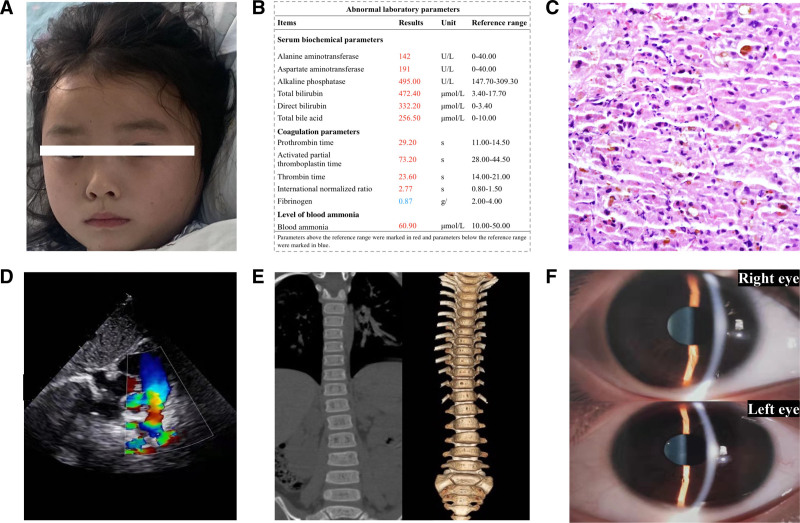
The result of clinical evaluation in the patient. (A) Characteristic facial features were not observed. (B) Abnormal laboratory parameters were detected. (C) Cholestasis was indicated by liver biopsy. (D) Echocardiography revealed pulmonary stenosis. (E) The CT scan and 3D reconstruction of spine showed no obvious abnormalities. (F) Posterior embryotoxon was not found through slit-lamp examination of eyes. CT = computed tomography.

The 5-year-old female with repeated jaundice during the first 5 years of life was admitted to the Lincang People’s Hospital on November 5, 2024, and then was transferred to Baoshan People’s Hospital one day later. Based on her parents’ description and our review of her previous medical records, we found that the patient was diagnosed with cholestasis at the age of 1 month in a township hospital but did not receive targeted therapy. After admission, detailed clinical examinations and genetic testing were performed on this patient. Characteristic facial features were not observed (Fig. 2A). Analysis of the serum biochemical parameters showed elevated levels of alanine aminotransferase (142.00 U/L), aspartate aminotransferase (191.00 U/L), alkaline phosphatase (495.00 U/L), total bilirubin (472.40 μmol/L), direct bilirubin (332.20 μmol/L), and total bile acid (256.50 μmol/L), suggesting intrahepatic cholestasis and liver dysfunction. Blood ammonia level (60.90 μmol/L) was increased. Meanwhile, coagulation parameters were found to be abnormal. In detail, the prothrombin time (29.20 seconds), activated partial thromboplastin time (73.20 seconds) and thrombin time (23.60 seconds) were significantly prolonged, and international normalized ratio (2.77) was increased, while the level of fibrinogen (0.87 g/L) was decreased (Fig. 2B). Cholestasis and pulmonary artery stenosis were observed in this patient via liver biopsy (Fig. 2C) and echocardiography (Fig. 2D), respectively. Additionally, the computed tomography scan and 3D reconstruction of spine (Fig. 1E) and slit-lamp examination of eyes (Fig. 2F) indicated no obvious abnormalities. The patient was clinically suspected to have ALGS according to the above clinical characteristics.

**Figure 3.**
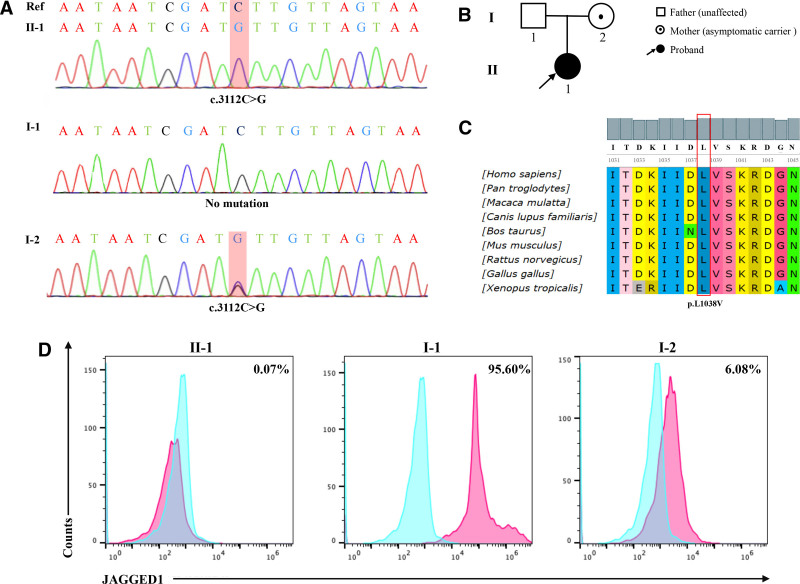
Molecular genetic characteristics. (A) The patient (II-1) was found to have a novel heterozygous *JAG1* missense mutation (c.3112C > G, p.L1038V) inherited form her mother (I-2), while the father (I-1) of this proband had no *JAG1* mutations. The mutation was indicated by the red rectangle. (B) The pedigree of this family showed that the patient (II -1) was the proband with a *JAG1* mutation, her mother (I-2) was an asymptomatic carrier of the same mutation, and her healthy father (I-1) was not found to have the *JAG1* mutation. (C) Evolutionary conservation analysis of amino acid 1038 in *JAG1* revealed that the mutated amino acid (L1038V) was located at a highly conserved position. (D) Flow cytometry indicated that JAGGED1 protein encode by *JAG1* was nearly unexpressed in the patient (II-1, 0.07%), faintly expressed in her mother (I-2, 6.08%) who is an asymptomatic mutation carrier, and normally expressed in her unaffected father (I-1, 95.60%). The peaks of blank control and subjects were labeled with blue and magenta, respectively.

Whole-exome sequencing (WES) with an on-target coverage of 98% and a mean sequencing depth of 127× was performed in the patient for exploring the underlying pathogenic mutation, which might be responsible for the clinical phenotype in this patient. The sequencing result revealed a novel heterozygous missense mutation in the exon 25 of the *JAG1* gene in the patient (II-1) (Fig. 3A). Specifically, a single nucleotide C-to-G substitution was found at nucleotide position 3112 of *JAG1* (c.3112C > G), causing a single amino acid alternation from leucine (L) to valine (V) at 1038th amino acid (p.L1038V). Subsequently, the validation of the novel *JAG1* c.3112C > G mutation (variant allele fraction: 23%) was performed using Sanger sequencing to exclude the possibility of false positive in WES, and the result confirmed that the mutation was inherited from her asymptomatic mother (I-2) with the same heterozygous *JAG1* variant and no mutation was detected in the healthy father of the patient (I-1) (Fig. 3A, B). According to the criteria from American College of Medical Genetics and Genomics, this novel mutation previously unreported in Human Genome Mutation Database Professional version was classified as a causative mutation. Meanwhile, this mutation was found to be highly conserved across multiple species (Fig. 3C), suggesting its potential importance to protein function. Furthermore, in order to further evaluate the effect of the novel mutation on the encoded protein JAGGED1, flow cytometry was subsequently employed. JAGGED1 was almost unexpressed in the patient (I-1, 0.07%) and faintly expressed in the mother (I-2, 6.08%) who is an asymptomatic carrier of the novel mutation (Fig. 3D). In contrast, a normal expression of this protein was showed in the unaffected father (I-1, 95.60%) (Fig. 3D). The result of flow cytometry indicated that the novel missense mutation changed the normal structure of *JAG1*, resulting in the failure of protein encoded by the mutant *JAG1* to bind to the normal Anti-JAGGED1 antibody (Abcam, ab273571, UK), and this failure was reflected as defects in the expression of wild-type JAGGED1. Combining the aforementioned clinical and molecular information, the patient was diagnosed with ALGS and this novel mutation in *JAG1* was thought to be the genetic etiology.

## 3. Discussion

The *JAG1* gene is widely expressed in multiple tissues, including the liver, heart, and gallbladder. As a key ligand in the Notch signaling pathway, JAGGED1 encoded by *JAG1* initiates downstream signaling by binding to the transmembrane Notch receptors, thereby involving in the regulation of cell proliferation, apoptosis, differentiation and playing a crucial role in the development of many organ systems.^[12,13]^ Thus, when *JAG1*, the fundamental component of the Notch pathway, is mutated, the Notch downstream cascade and cell fates are affected, thereby the normal development and function of the various organs may be disrupted.^[14,15]^ In this study, we identified a novel heterozygous missense mutation in the *JAG1* gene in a Chinese family and the proband presented with chronic cholestasis and pulmonary artery stenosis. Such a patient would be easily missed or misdiagnosed in the absence of genetic testing.

ALGS has protean clinical manifestations and not all affected individuals presented with the classic phenotype. According to the previous studies, 89% of ALGS cases had cholestasis,^[16]^ 94% had congenital cardiac disease,^[17]^ 70% to 96% had specific facial characteristics (triangular contour of the face, prominent forehead, prominent forehead, straight nose with bulbous tip, deep-set eyes and pointed chin),^[18]^ 78% to 89% had posterior embryotoxon,^[19,20]^ 33% to 87% had butterfly vertebrae,^[5,16]^ and some patients may also suffer from renal and vascular abnormalities.^[3]^ The above perspectives can adequately explain the following phenomenon: the patient in the present study did not show other ALGS-related abnormalities except for chronic cholestasis and pulmonary stenosis. Another intriguing phenomenon in this work is that ALGS phenotype was present in our patient, but not in her mother who carried the same causative *JAG1* mutation (c.3112C > G, p.L1038V). This again reflected the variable expressivity and phenotypic penetrance of ALGS. In a retrospective study on a cohort of *JAG1* mutation carriers from Children’s Hospital of Philadelphia, 23 of 53 *JAG1* mutation-positive relatives of 34 probands from 34 families had only 1 or 2 clinical characteristics of ALGS and 2 of 53 were asymptomatic carriers.^[2]^ Even within a single family, different clinical situations could be observed. Based upon a previous literature reported by Guo et al,^[21]^ a female proband showed an ALGS phenotype including cholestasis, posterior embryotoxon, growth restriction and facial dysmorphia, her sister had similar manifestations except growth restriction, whereas the mother of these 2 patients was healthy. Hence, some researchers speculated that the variable expressivity and penetrance of ALGS were closely related to some potential genetic modifiers, such as Thrombospondin2 gene, which was identified as a candidate modifier of ALGS-related live disorders.^[22]^ But overall, the data of genetic modifiers are limited and more research is needed.

Moreover, it is noteworthy that hepatic abnormality is one of the major causes of death in ALGS.^[2]^ Because of persistent cholestasis, approximately 21% of ALGS individuals progressed to severe liver disease requiring liver transplantation.^[19]^ In a recent multicenter study, the transplant‐free survival rate of ALGS with cholestasis was 24% at the age of 18.5 years.^[23]^ Severe liver dysfunction, abnormal coagulation and low hemoglobin was detected in our patient at the first day of admission, which subsequently showed no significant improvement despite 2-day supportive treatments therapy (blood transfusion, vitamin K1 supplementation, application of liver-protection drugs). On the third day of hospitalization, the patient’s guardians have been informed of the patient’s condition and the necessity of blood purification. We also advised that if a diagnosis of ALGS were fully confirmed (despite high clinical suspicion of ALGS based on clinical features at admission, WES results were not available until 19 days later) and accompanied by severe hepatic failure, liver transplantation could be considered as a crucial treatment option. However, the guardians refused the above potential treatment schemes, chose to continue with supportive care, and requested discharge on hospital day 7. In addition to hepatic abnormalities, serious heart disease was considered as another important factor related to mortality in ALGS. The 6-year survival rate in ALGS patients with cardiac disease was 40%, which was significantly lower than that (95%) in ALGS patients without cardiac abnormalities.^[24]^ Although our patient had pulmonary artery stenosis, no relevant clinical symptoms were observed in her. Periodic reexamination should be performed for cardiac health monitoring. However, regrettably, the guardians gave up further examinations and treatments for the patient and asked to be discharged. Soon after, the patient died at home due to deterioration of her condition. Frankly, in terms of ALGS treatment, the current therapies are supportive and directed to address specific clinical manifestations, rather than the underlying pathophysiology of disordered Notch signaling pathway.^[25,26]^ Meanwhile, due to the absence of genotype–phenotype relationships in ALGS, it is still remained unknown whether the mutation location of *JAG1* can play a positive role in personalized therapy for ALGS. Theoretically, new therapeutic approaches in the future may involve modulation of Notch pathway signaling, cell-based therapies, or correction of specific mutations in vitro or in vivo. For example, one of the theoretical methods would be to increase expression of the normal *JAG1* or *NOTCH2* allele by delivering mRNA or by altering transcriptional regulation. Direct delivery of the JAGGED1 ligand has been shown to effectively facilitate fracture healing in a mouse model.^[27]^ Besides, there are studies on inducing pluripotent stem cells by using CRISPR/Cas9 technology in human to correct mutations in other inherited diseases in vitro.^[28]^ In general, these approaches are under investigation in animal models or in vitro, and have great potential as viable therapeutic interventions for ALGS.

Despite the findings in this report, there were 3 main limitations in this study. The first key limitation of this study was that our observation was based on a single patient, and we were unable to recruit additional cases to more comprehensively investigate the potential association between this novel mutation and the clinical phenotype. The second was that we did not perform further molecular biology or animal experiments to further verify the pathogenicity of the novel mutation, conducting a more in-depth exploration of its pathogenic mechanism. Thirdly, due to the unavailability of the blood samples, we failed to perform genetic testing on the more family members other than the proband’s parents. These limitations highlight the important avenues for our future work in this flied.

In summary, we utilized WES to identify a novel causative *JAG1* mutation (c.3112C > G, p.L1038V), which was confirmed as the pathogenic molecular cause for the ALGS phenotype in our patient. This finding broadened the mutational spectrum of the *JAG1* gene and provided clues for further analysis of the complex genotype-phenotype correlation in ALGS. Simultaneously, our report also reflected the importance of molecular diagnosis for diseases with highly variable phenotypes, such as ALGS. Additionally, potential genetic modifiers of ALGS should be further investigated in the future for a better understanding of the precise molecular mechanism of ALGS.

## Acknowledgments

The authors sincerely thank the patient and her family members for their participation and support.

## Author contributions

**Conceptualization:** Zongdan Zhang, Chuangfeng He.

**Data curation:** Zongdan Zhang, Xuezu Zhang.

**Formal analysis:** Zongdan Zhang, Xuezu Zhang.

**Investigation:** Zongdan Zhang, Xuezu Zhang, Chuangfeng He.

**Methodology:** Chuangfeng He.

**Visualization:** Zongdan Zhang.

**Writing – original draft:** Zongdan Zhang, Xuezu Zhang.

**Writing – review & editing:** Chuangfeng He.
